# Systematic Genomic and Clinical Analysis of Severe Acute Respiratory Syndrome Coronavirus 2 Reinfections and Recurrences Involving the Same Strain

**DOI:** 10.3201/eid2801.211952

**Published:** 2022-01

**Authors:** Cristina Rodríguez-Grande, Luis Alcalá, Agustín Estévez, Pedro J. Sola-Campoy, Sergio Buenestado-Serrano, Carolina Martínez-Laperche, Víctor Manuel de la Cueva, Roberto Alonso, Cristina Andrés-Zayas, Javier Adán-Jiménez, Carmen Losada, Carla Rico-Luna, Iñaki Comas, Fernando González-Candelas, Pilar Catalán, Patricia Muñoz, Laura Pérez-Lago, Darío García de Viedma, on behalf of the Gregorio Marañón Microbiology-ID COVID 19 Study Group

**Affiliations:** Gregorio Marañón General University Hospital, Madrid, Spain (C. Rodríguez-Grande, L. Alcalá, A. Estévez, P.J. Sola-Campoy, S. Buenestado-Serrano, C. Martínez-Laperche, V.M. de la Cueva, R. Alonso, C. Andrés-Zayas, J. Adán-Jiménez, C. Losada, C. Rico-Luna, P. Catalán, P. Muñoz, L. Pérez-Lago, D. Garcia de Viedma);; Instituto de Investigación Sanitaria Gregorio Marañón, Madrid (C. Rodríguez-Grande, L. Alcalá, A. Estévez, P.J. Sola-Campoy, S. Buenestado-Serrano, C. Martínez-Laperche, V.M. de la Cueva, R. Alonso, C. Andrés-Zayas, J. Adán-Jiménez, C. Losada, C. Rico-Luna, P. Catalán, P. Muñoz, L. Pérez-Lago, D. García de Viedma);; CIBER Enfermedades Respiratorias, Madrid (L. Alcalá, R. Alonso, P. Catalán, P. Muñoz, D. García de Viedma);; Instituto de Biomedicina de Valencia-CSIC, Valencia, Spain (I. Comas);; CIBER Salud Pública, Madrid (I. Comas, F. González-Candelas); J; oint Research Unit “Infection and Public Health” FISABIO-University of Valencia, Institute for Integrative Systems Biology, Valencia (F. González-Candelas);; Universidad Complutense de Madrid, Madrid (P. Muñoz)

**Keywords:** COVID-19, coronavirus disease, SARS-CoV-2, severe acute respiratory syndrome coronavirus 2, viruses, respiratory infections, zoonoses, reinfection, recurrence, variant of concern, variant of interest, Madrid, Spain

## Abstract

Estimates of the burden of severe acute respiratory syndrome coronavirus 2 reinfections are limited by the scarcity of population-level studies incorporating genomic support. We conducted a systematic study of reinfections in Madrid, Spain, supported by genomic viral analysis and host genetic analysis, to cleanse laboratory errors and to discriminate between reinfections and recurrences involving the same strain. Among the 41,195 cases diagnosed (March 2020–March 2021), 93 (0.23%) had 2 positive reverse transcription PCR tests (55–346 days apart). After eliminating cases with specimens not stored, of suboptimal sequence quality, or belonging to different persons, we obtained valid data from 22 cases. Of those, 4 (0.01%) cases were recurrences involving the same strain; case-patients were 39–93 years of age, and 3 were immunosuppressed. Eighteen (0.04%) cases were reinfections; patients were 19–84 years of age, and most had no relevant clinical history. The second episode was more severe in 8 cases.

Since the first description of a severe acute respiratory syndrome coronavirus 2 (SARS-CoV-2) reinfection on August 24, 2020, in a patient from Hong Kong (*1*) who acquired a second infection after having traveled to Europe, several reports have described other individual reinfection cases in different countries. These cases suggest the lack of a common reinfection pattern, with a variety of intervals between episodes, severity of episodes, clinical history, etc (*2*–*5*).

Genomic viral analysis has been applied to determine within-host SARS-CoV-2 evolution in patients with persistent infection (*6*,*7*) but has not been used in the same way to analyze coronavirus disease (COVID-19) recurrences involving the same strain. The scant available reports focus primarily on clinical descriptions (*8*,*9*), only some of which are supported by detailed genomic analysis (*10*).

Although a reasonable number of studies have analyzed individual COVID-19 recurrences in detail, population-level studies addressing this issue more systematically are lacking. We present a systematic analysis of all COVID-19 recurrences diagnosed at a tertiary hospital in Madrid, Spain (320,956 case-patients, 11.3% of the total Madrid population), over a 12-month period. Our analysis was supported by genomic viral analysis, cleansing of laboratory errors by host genetic analysis, consideration of both reinfections and recurrences involving the same strain, and integrating clinical features of the cases.

## Materials and Methods

### Patients and Methods

The study period was March 2020−March 2021. The cases selected for study were required to have 2 sequential positive reverse transcription PCR (RT-PCR) tests taken >45 days apart with >1 negative RT-PCR between positive tests. When the interepisode interval was >120 days and a different lineage was involved in each episode, the negative RT-PCR between episodes was not obligatory.

### Specimens

The specimens corresponded to the remnants of nasopharyngeal swabs taken for diagnostic purposes. Specimens were stored at −70°C until analysis.

### Clinical Data

The baseline characteristics, clinical and laboratory parameters at COVID-19 diagnosis, and outcomes of patients were obtained from their electronic medical records. The study was approved by the ethical research committee of Gregorio Marañón Hospital, Madrid (REF: MICRO.HGUGM.2020–042).

### Diagnostic Tests

#### SARS-CoV-2 RT-PCRs and Serology

We extracted and purified viral RNA from 300 μL of nasopharyngeal exudates with the KingFisher instrument (ThermoFisher Scientific, https://www.thermofisher.com). This process was followed by RT-PCR using the TaqPath COVID-19 CE-IVD RT-PCR kit (ThermoFisher Scientific), which targets open reading frame 1ab, nucleoprotein, and spike genes. We performed serum antibody determinations by specific quantitative detection of SARS-CoV-2 IgG by using a chemiluminescent microparticle immunoassay on the ARCHITECT system (SARS-CoV-2 IgG II Quant Reagent Kit; Abbott Laboratories, https://www.abbott.com).

### Whole-Genome Sequencing

We used 11 μL of RNA as template for reverse transcription using Invitrogen SuperScript IV reverse transcription and random hexamers (both ThermoFisher Scientific). We performed whole-genome amplification of the coronavirus with an Artic_nCov-2019_V3 panel of primers (Integrated DNA Technologies, Inc., https://www.idtdna.com) (https://artic.network/ncov-2019) and Q5 Hot Start DNA polymerase (New England Biolabs, https://www.neb.com). We prepared libraries by using the Nextera DNA Flex Library Preparation Kit (Illumina, https://www.illumina.com), following the manufacturer’s instructions.

Libraries were quantified with a Quantus Fluorometer (Promega, https://www.promega.com) before being pooled at equimolar concentrations (4 nM). We then sequenced them in pools of <17 libraries on the Miseq system (Illumina Inc.) with the MiSeq Reagent Micro kit version 2 (2×151 bp) or in pools of <96 libraries with the MiSeq Reagent (2×201 bp).

Sequences above the GISAID thresholds were deposited at GISAID (https://www.gisaid.org; Appendix 1 Table). An in-house analysis pipeline was applied on the sequencing reads (https://github.com/pedroscampoy/covid_multianalysis). In brief, the pipeline involves the following 4 steps: 1) removal of human reads with Kraken (*11*); 2) preprocessing and quality assessment of fastq files using fastp version 0.20.1 (*12*) (arguments: –cut tail, –cut-window-size, –cut-mean-quality, –max_len1, –max_len2) and fastQC version 0.11.9 (https://www.bioinformatics.babraham.ac.uk/projects/fastqc); 3) mapping with BWA version 0.7.17 (*13*) and variant calling using IVAR v1.2.3 (*14*), using the Wuhan-1 SARS-CoV-2 sequence (GenBank accession no. NC_045512.2) as reference; and 4) calibration of occasional low coverage positions using joint variant calling. When necessary, we analyzed informative noncovered positions by standard Sanger sequencing by using the corresponding flanking primers from the ARTIC set.

Reinfections were considered when we detected a higher than expected number of single-nucleotide polymorphisms (SNPs) between the episodes (considering the standard estimation of 1 SNP/2 weeks) or a distribution of SNPs between the episodes consistent with independent evolutionary paths (SNPs present in the first episode but absent in the second episode and vice versa), or different variants or lineages involved in each episode or involvement in the second episode of a strain or variant that was not circulating in the population when the patient had the first episode. Recurrences were considered to involve the same strain when 0–1 SNPs were identified between the sequences from each episode.

### Short Tandem Repeat Analysis

For human identity testing, we applied short tandem repeat (STR) PCR using the Mentype Chimera PCR amplification kit (Biotype, https://www.biotype.de) on the specimens used for SARS-CoV-2 genome sequencing. We examined 12 noncoding STR loci and the gender-specific amelogenin locus, labeled with 3 different dyes (6-FAM, BTG, or BTY). The selected loci had a very high rate of heterozygosity and balanced allelic distribution (*15*). We performed PCR with 0.2–1 ng of genomic DNA using the Mentype Chimera PCR amplification kit (Biotype), the GeneAmp PCR System 9700 Thermal Cycler, followed by capillary electrophoresis on a Genetic Analyzer 3130*xl* (both ThermoFisher Scientific), as recommended by the manufacturer.

## Results

**Figure 1:**
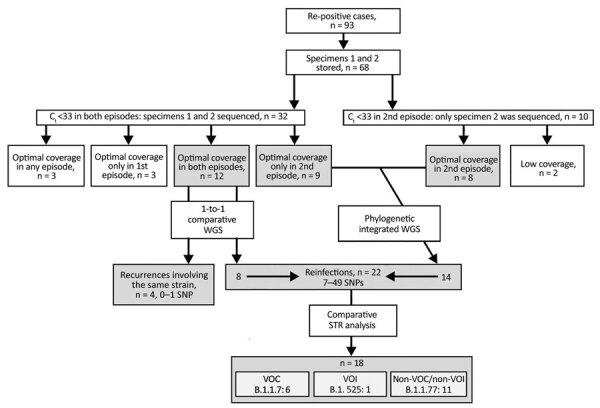
Flow diagram of the analysis and selection criteria for severe acute respiratory syndrome coronavirus 2 re-positive cases, Madrid, Spain, March 2020–March 2021. Re-positive cases were those that had 2 sequential positive RT-PCR tests taken >45 days apart with >1 negative RT-PCRs between the positive tests. C_t_, cycle threshold; SNP, single-nucleotide polymorphism; STR, short tandem repeat; VOC, variant of concern; VOI, variant of interest; WGS, whole-genome sequencing.

The criteria for selecting SARS-CoV-2–positive cases for the study was 2 sequential positive RT-PCR tests taken >45 days apart with >1 negative RT-PCRs between the positive tests. Of the 41,195 cases diagnosed during the study period (March 2020–March 2021), 93 (0.23%) fulfilled these criteria, with positive specimens taken 55–346 days apart (Figure 1). We classified these cases as re-positive. Two specimens had been stored for each of 68 (73%) of the 93 re-positive cases, and of these, 32 (34%) were suitable for sequencing and comparison of sequences because cycle threshold (C_t_) values for both positive specimens were <33 (Figure 1). The sequencing quality parameters of the 2 specimens were above the recommended threshold for a robust SNP calling (>90% of the genome with >30× coverage depth) in only 12 cases (29%). In another 17 cases, only 1 of the 2 specimens offered sequences of sufficient quality (Figure 1).

### Recurrences Involving the Same Strain

After comparing the SNPs called in the sequences from the sequential episodes of the 12 re-positive cases, 4 (0.01% of total diagnosed cases) were classified as recurrences involving the same strain (Table 1) (0–1 SNPs between them; 3 belonged to A.5 lineage and 1 to Z.1 lineage [parental lineage: B.1.177.50]). Time between episodes ranged from 55 to 114 days, and C_t_ values for the second episode were consistent with active infection (C_t_ 19–28). All had 1 negative result between the positive SARS-CoV-2 RT-PCR test, and 1 also had a second intermediate negative test.

**Table 1:** Patient data and characteristics for both episodes of SARS-CoV-2 infection in recurrences involving the same strain, Madrid, Spain, March 2020–March 2021*

Pt	Age, y/sex	Underlying conditions	PCR date, 1st/2nd episode	PCR C_t_ value–N_2_ gene, 1st/2nd episode	Interinfection period, d	Symptoms, 1st/2nd episode†	Serologic results, 1st/2nd episode (AU/mL)	SARS-CoV-2 variant, 2nd episode
1	68/M	HT	2020 Aug 12/2020 Dec 4	15/28	114	Asymptomatic/ asymptomatic	+ (56.8)/+ (76.8)	Z.1
2	70/F	HBP, obesity	2020 Mar 25/2020 Jun 10	31/27	77	Bilateral pneumonia/ dyspnoea	NA/+ (1,967.1)	A.5
3	93/F	HBP, CKD	2020 Apr 17/2020 Jun 11	31/19	55	Diarrhea/NA	–/NA	A.5

4

39/F

BMT

2020 Mar 10/2020 Jun 19

17/27

101

Bilateral pneumonia/ diarrhea, fever

+ (415.4)/+ (636.3)

A.5

*BMT, bone marrow transplant; CKD, chronic kidney disease; HBP, high blood pressure; HT, heart transplant; N_2_, nucleocapsid; NA, not available; Pt, patient; SARS-CoV-2, severe acute respiratory syndrome coronavirus 2. †The definition of the severity of the patients has been organized according to the following criteria: Mild—general malaise, cough, diarrhea, headache, fever, anosmia, dysgeusia, myalgia, rhinorrhea; moderate—previous symptoms plus dyspnea, mild respiratory failure, or unilateral pneumonia, severe—previous symptoms plus bilateral pneumonia.

The 4 patients ranged from 39 to 93 years of age; underlying conditions were 1 heart transplant, 1 bone marrow transplant, 1 case of chronic renal insufficiency, and 1 case of obesity and high blood pressure (Table 1). Of the patients with underlying conditions, 3 had a clinical history of some degree of chronic immunosuppression: case-patient 1 underwent a heart transplant in June 2020 and was being treated with prednisone and mycophenolate, case-patient 3 had chronic kidney disease, and case-patient 4 underwent a bone marrow transplant in 2019 and was receiving treatment with sirolimus and ruxolitinib. Case-patient 2 had no known immunosuppression. Case-patients 1 and 4 seroconverted after the first SARS-CoV-2–positive episode (Table 1). Serologic testing was not available for case-patient 2, and case-patient 3 had a negative serologic result but was measured soon after the primary infection. For the second SARS-CoV-2 infection, case-patients 1, 2, and 4 seroconverted; results of serologic testing were not available for case-patient 3 (Table 1). In 2 cases, the second episode was milder in severity. In another case, both episodes were asymptomatic; for the remaining case-patient, who had a mild first episode, data were not available for the second infection. Two case-patients were asymptomatic between the 2 episodes, and the other 2 experienced asthenia and general malaise.

### Reinfections

**Figure 2:**
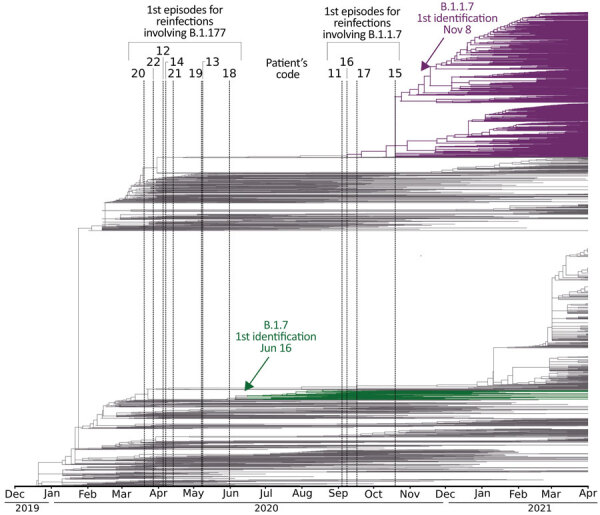
Global dating of the first emergence of severe acute respiratory syndrome coronavirus 2 variants identified in reinfections, Madrid, Spain, March 2020–March 2021, with available sequences only for the second specimen (patients 11–22). The phylogeny includes the 3,339 genomes from Nextstrain (https://nextstrain.org), extracted from the Europe-focused subsampling, through April 2021. Dates of the first episodes of cases are indicated with vertical lines. Dates for global emergence for the variants involved in their second episodes (B.1.1.7 and B.1.177) are indicated with an arrow and correspond to their first descriptions in Spain (as documented in GISAID, https://www.gisaid.org): for B.1.177, June 16, 2020 (hCoV-19/Spain/IB-IBV-99010764/2020, accession no. EPI_ISL_691664) and for B.1.1.7, November 8, 2020 (hCoV-19/Spain/VC-IBV-98012610/2020, accession no. EPI_ISL_1060510). Only reinfection cases finally validated by short tandem repeat host analysis are included.

In 8 of the remaining re-positive cases, we identified 7–49 different SNPs between the sequences from the 2 sequential positive specimens, which indicated that they were reinfections (Table 2; Figure 1; Appendix 2 Figure). In addition to the standard approach to identifying reinfections (i.e., direct comparison of SNPs between SARS-CoV-2 sequences obtained in 2 sequential episodes), we also followed an alternative approach (*16*) using a population-based integrated phylogenetic approach to demonstrate that the second episode involved a strain that was not circulating in the population during the patient’s first episode. To apply this alternative strategy, we needed sequencing data only from the second episode of COVID-19. Therefore, we recovered the 9 cases from the second episode providing optimal sequence coverage that had been ruled out for 1-to-1 SNP comparisons (Figure 1). We were also able to add a further 8 cases with optimal sequences out of 10 cases with C_t_ values <33 in the second episode that had previously been ruled out for comparative sequencing (Figure 1). In 14 cases, we identified SARS-CoV-2 variants (9 B.1.177 and 5 B.1.1.7) with dates of emergence in our population after these patients experienced their first episodes (Figure 2). The first description in Spain for B.1.177 was June 16, 2020 (hCoV-19/Spain/IB-IBV-99010764/2020; GISAID accession no. EPI_ISL_691664) and for B.1.1.7 was November 8, 2020 (hCoV-19/Spain/VC-IBV-98012610/2020; accession no. EPI_ISL_1060510). This information indicates that the variants involved in these patients’ second episodes were not circulating in Spain at the time of their first episodes and therefore correspond to reinfections.

**Table 2:** SARS-CoV-2 variants and SNP distances involved in reinfections identified by 1-to-1 whole-genome sequencing comparison for patients in Madrid, Spain, March 2020–March 2021*

Patient	Specimen 1	Specimen 2	No. SNPs†
5	B	B.1.177	22
6	B.1.258	B.1.177	24
7	B.1.177	B.1.177	7
8	A	B.1.525	40
9	W.4	B.1.1.7	47
10	W.4	B.1.1.7	49

*The 2 reinfections not confirmed (short tandem repeat host analysis revealed that the 2 sequential specimens belonged to different patients) are not included. SARS-CoV-2, severe acute respiratory syndrome coronavirus 2; SNP, single-nucleotide polymorphism. †Including insertions/deletions.

We subjected the 22 total reinfections assigned according to the standard or alternative phylogenetic approaches to a final validation to demonstrate that the specimens in the first and second episodes belonged to the same host and to rule out erroneous assignment of reinfections as a result of incorrect labeling or handling of samples. STR genetic analysis identified 2 pairs of specimens with genetic differences, whereas STR analysis of 2 specimens from 2 cases did not yield interpretable results; we eliminated all 4 cases from the study, leading to final validation of 18 reinfections (0.04% of total diagnosed cases and 81.82% of initially suspected reinfections by viral genomic analysis).

The positive specimens from the 18 reinfection cases validated by host genetic analysis were taken 116–342 days apart. Of these 18 cases, 6 reinfections involved the B.1.1.7 (Alpha) variant of concern, 1 involved the B.1.525 variant of interest, and the remaining 11 cases involved the B.1.177 variant (neither variant of concern nor variant of interest).

The age range for reinfected cases was 19 to 84 years of age. Most (13/18) had no relevant clinical antecedents (Table 3), and of those with underlying conditions, we highlight 1 renal transplant, 1 case-patient with asthma, 1 with chronic kidney disease, and 1 with autoimmune disease. In those for whom serologic data were available for the first and second episodes, SARS-CoV-2 serologic test results were positive in 2/9 first episode cases and 11/11 second episode cases (Table 3). For the first episode, 6 case-patients were asymptomatic, 6 had mild symptoms, 6 were moderately symptomatic, and no cases were severe. The second episode was mild in 11 cases, and only 1 case-patient was asymptomatic. Comparing the symptoms for the sequential episodes, the second episode was more severe in 8 cases (bilateral pneumonia occurred in 3 case-patients); symptoms were milder in 1 case and equivalent to the first episode in the remaining cases.

**Table 3:** Patient data and characteristics for both episodes of SARS-CoV-2 infection in cases of reinfection, Madrid, Spain, March 2020–March 2021*

Pt	Age, y/sex	Underlying conditions	PCR date, 1st/2nd episode	PCR C_t_ value–N_2_ gene, 1st/2nd episode	Interinfection period, d	Symptoms, 1st/2nd episode†	Serologic results, 1st/2nd episode (AU/mL)	SARS-CoV-2 variant, 2nd episode
5	54/F	None	2020 Mar 20/ 2021 Jan 12	32/24	298	Cough, myalgia, anosmia, dysgeusia, dyspnea/ rhinorrhea, headache, anosmia, dysgeusia	+ (647.8)/+ (35,823.2)	B.1.177
6	67/M	HBP	2020 Mar 28/ 2021 Jan 21	19/18	299	Dyspnea, fever, general malaise/fever, mild respiratory failure	–/+ (>40,000)	B.1.177
7	31/M	None	2020 Aug 1/ 2020 Dec 25	32/17	146	Asthenia/cough, rhinorrhea	–/+ (336.4)	B.1.177
8	18/M	CKD	2020 Mar 18/ 2021 Feb 23	21/19	342	Asymptomatic/ rhinorrhea	+ (473.8)/+ (4,122.2)	B.1.525
9	50/M	None	2020 Oct 18/ 2021 Feb 11	32/20	116	Asymptomatic/ cough, asthenia, bilateral pneumonia	NA/NA	B.1.1.7
10	23/M	None	2020 Aug 21/ 2021 Mar 10	26/28	201	Asymptomatic/ cough, rhinorrhea	NA/NA	B.1.1.7
11	19/F	None	2020 Sep 3/ 2021 Mar 16	NA/19	194	Asymptomatic/ general unrest, rhinorrhea, cough	NA/NA	B.1.1.7
12	54/F	Asthma, depression	2020 Apr 4/ 2020 Aug 22	30/22	140	Dyspnea, fever, cough/bilateral pneumonia	–/+ (14,307.4)	B.1.177
13	84/F	CKD, HBP, RT	2020 May 7/ 2020 Oct 24	NA/22	170	Asthenia, dyspnea/ bilateral pneumonia	–/+ (18,088.1)	B.1.177
14	42/F	None	2020 Apr 6/ 2020 Oct 20	NA/19	197	General malaise/cough	–/+ (212.9)	B.1.177
15	49/F	None	2020 Oct 18/ 2021 Feb 11	33/14	116	General malaise/ myalgia, fever	NA/NA	B.1.1.7
16	20/F	None	2020 Sep 7/ 2021 Feb 5	27/19	151	Cough/ asymptomatic	NA/NA	B.1.1.7
17	39/F	None	2020 Sep 15/ 2021 Feb 9	30/16	147	Dyspnea, fever/ cough, dyspnea, myalgia	NA/NA	B.1.1.7
18	29/F	None	2020 May 30/ 2021 Jan 23	39/23	238	Asymptomatic/ diarrhea, cough, headache	NA/+ (275.8)	B.1.177
19	47/F	None	2020 May 6/ 2020 Sep 23	36/32	140	Unilateral pneumonia/ fever, anosmia	NA/+‡	B.1.177
20	55/F	Autoimmune	2020 Mar 18/ 2021 Jan 25	33/26	313	Asymptomatic/ general malaise	–/+ (1,345.4)	B.1.177
21	73/F	None	2020 Apr 12/ 2021 Feb 4	34/17	298	HBP/NA	NA/NA	B.1.177
22	58/F	None	2020 Mar 26/ 2021 Jan 26	32/28	306	Headache/general malaise	–/+ (56.4)	B.1.177

*CKD, chronic kidney disease; HBP, high blood pressure; N_2_, nucleocapsid; NA, not available; pt, patient; RT, renal transplant; SARS-CoV-2, severe acute respiratory syndrome coronavirus 2. †The definition of the severity of the patients has been organized according to the following criteria: Mild—general malaise, cough, diarrhea, headache, fever, anosmia, dysgeusia, myalgia, rhinorrhea; moderate—previous symptoms plus dyspnea, mild respiratory failure, or unilateral pneumonia, severe—previous symptoms plus bilateral pneumonia. ‡Test performed in another center; numeric data not available.

## Discussion

Since the first description of a SARS-CoV-2 reinfection (*1*), many reports have been published documenting single cases of reinfection (*2*–*5*) and demonstrating the wide variety of ages, clinical backgrounds, and severity among episodes (*17*). According to the European Centre for Disease Prevention and Control (ECDC), in the 12 European Union countries that reported cases, 1,887 likely reinfections in 2020 and 691 likely reinfections from January–February 2021 were under investigation (*18*).

Despite the large number of reports communicating SARS-CoV-2 reinfections, they are rare, although estimates of the true impact are limited by the scarcity of larger population-level studies. A nationwide study performed in Denmark (*19*) concluded that 0.65% of SARS-CoV-2–positive cases during the first COVID-19 wave had a second positive test in the second wave, and that this percentage increased to 3.27% in those with a negative result in the first wave. These data allowed Hansen et al. (*19*) to infer that protection against repeat infection in those who had natural immunity from previous SARS-CoV-2 infection was 80.5%, decreasing to 47.1% among persons >65 years of age.

Other studies have tried to go beyond the reporting of single cases by offering data on the frequency of SARS-CoV-2 reinfections in different countries; results range from 0.14 to 2.11% (*19*–*24*). However, in all these studies, the assignment of reinfections was supported only by sequential positive RT-PCR results, which means that, strictly speaking, these re-positive SARS-CoV-2 infections were considered suspected reinfections (*22*) without determining whether they were recurrences involving the same strain, reinfections, persistent cases, or testing errors (*25*). Assigning re-positive cases to 1 of the above categories is only possible when whole sequencing data are also included in the analysis.

The aim of our study was to overcome these limitations by enhancing the robustness of a systematic study of all COVID-19 cases diagnosed in our population, with the added value of a refined genomic analysis and considering both viral genomic analysis and host genetic analysis. This design makes it possible to precisely assign recurrences involving the same strain and reinfections and to cleanse test errors, in short of being able to offer solid data on the actual burden of these events in our population. Equivalent efforts should be made to study the impact of these events in other communities.

The percentage of re-positive cases we observed before genomic analysis (0.23%) is similar to that observed in other settings (*26*,*27*). To consider a case re-positive, we established a threshold of 45 days between 2 SARS-CoV-2–positive RT-PCR tests with >1 intermediate negative RT-PCR result, although in 69 of our 93 re-positive cases (74.2%), the episodes were >90 days apart.

Despite efforts to store specimens since the beginning of the pandemic, in 27% of the 93 re-positive cases, >1 of the 2 specimens were not available in our biobank, illustrating a main challenge of documenting reinfections (*17*). In addition to loss of cases, a second challenge was obtaining high-quality sequencing data, which was achieved in only one third of the cases with available specimens. In our experience processing recent specimens, the percentage of specimens with C_t_ values <33 that yielded suboptimal sequencing data was much lower (7%–10%). This experience serves as a cautionary warning of the potential deterioration of valuable remaining diagnostic specimens, even at −80° C, for future studies.

After comparative viral genomic analysis, identification of recurrences involving the same strain accounted for a reduction of 18.2%, and host genetic analysis a further 9.1% reduction (because specimens came from different persons), in the number of re-positive cases that would otherwise have been wrongly assigned as reinfections. On the basis of this finding, we also eliminated from the study another 2 cases with suboptimal results in the host genetic analysis, which did not enable us to draw conclusions. The dramatic increase in laboratory workload during the successive waves of COVID-19 infection likely led to mistakes in labeling samples or aliquoting. However, only a few studies that focused on documenting SARS-CoV-2 reinfections considered ruling out mislabeling of specimens by host genetic analysis (*2*,*28*). Our data indicate that a proportion of reinfections are more likely to be misassigned if genomic rigor is applied only to viral analysis and not to host analysis.

Of note, we used 2 approaches to assess reinfections. The first was the standard direct comparison of SARS-CoV-2 sequences, which revealed 6 reinfections, all but 1 differing by >20 SNPs (above the 2 SNPs/month estimated for SARS-CoV-2 evolution). The remaining reinfection differed by 7 SNPs, although the 7 differential SNPs were distributed in 3 SNPs that were specific to the first episode and not found in the second episode, and another 4 SNPs that were identified in the second episode but not in the first episode. This distribution of SNPs demonstrates that the second strain could not have evolved from the first one, consistent with reinfection. After the standard 1-to-1 comparative approach to identify reinfections, we applied a second alternative approach (*16*), based on a population-based integrated phylogenetic approach, to demonstrate that the strain involved in the reinfection had not yet emerged in our population at the time of the patient’s first episode. This alternative approach, in which we identified 12 additional reinfections, supports the need to expand the criteria for assigning SARS-CoV-2 reinfections, as the ECDC (*29*) did when it accepted the use of whole-genome sequencing to document reinfections by demonstrating that the strain involved in the reinfection was clustered with other strains circulating at the site of exposure (*29*). Considering the difficulties of storing all remaining specimens during the pandemic because of the high diagnostic workload, the alternative phylogenetic approach applied in this study could pave the way for more extensive documentation of the actual magnitude of reinfections in different populations.

A systematic review (*25*) concluded that reinfections were more likely to correspond to re-positive cases with a second positive RT-PCR >3 months after the first episode. Our reinfection data are consistent with this observation, because the time between episodes ranged from 116 to 346 days. Our data would fit the recent definition of a reinfection case by the ECDC (*18*), which establishes a 90-day threshold for reinfection to be considered.

The fact that most reported reinfections occurred >3 months after the first episode suggests the progressive decline in antibodies after a first infection plays a likely role. Unfortunately, in most studies, serologic data for first infections are lacking, which limits the documentation of this hypothesis. In our study, only 2 of 9 cases for which serologic data were available had positive SARS-CoV-2 serologic results, whereas all 11 seroconverted after the reinfection episode. Our data point to the lack of immune response mounted after the first infection being a more likely explanation for reinfection than a progressive decline in antibodies.

With respect to differences in severity between the first and second episodes in SARS-CoV-2 reinfections, situations vary widely (*17*). In our study, the second episode was generally more severe; we noted 6 asymptomatic, 6 mild, 6 moderate, and no severe first episodes versus 1 asymptomatic, 11 mild, 2 moderate, and 3 severe second episodes.

Not all re-positive cases >3 months after first infection should be assumed to correspond to reinfection. In our study, of the 4 recurrences identified that involved the same strain, 2 also occurred within this period, whereas the remaining 2 occurred 55 and 77 days after the first episode, beyond the threshold proposed as highly suggestive of nonreinfections (*25*,*30*).

SARS-CoV-2 recurrences involving the same strain have attracted much less attention than reinfections, possibly because of the lack of genomic resolution in most studies addressing reinfections with population-level values. Our data indicate that 18.2% of SARS-CoV-2 re-positive cases corresponded to recurrences involving the same strain, which would otherwise have been mislabeled as reinfections if genomic viral analysis had not been included. The second episode was equivalent or milder in terms of severity. Of recurrences involving the same strain, 3 corresponded to patients with some degree of immunosuppression (renal transplantation, bone marrow transplantation, and chronic kidney disease). The very few cases of SARS-CoV-2 recurrences involving the same strain reported in other studies supported by genomic analysis also occurred in immunosuppressed patients (D.A. Molina, unpub. data, https://www.researchsquare.com/article/rs-92286/v1; *8*).

The robustness of our study’s systematic design was coupled with the value of its methodological refinement, which integrated genomic viral analysis and host genetic analysis. This design enabled us to cleanse data by eliminating laboratory errors and to offer precise data about the true burden and clinical features of SARS-CoV-2 reinfections and recurrences involving the same strain. We performed our analysis before the emergence of most SARS-CoV-2 variants of concern. Therefore, this study constitutes a valuable reference for forthcoming comparative studies addressing the burden of reinfections and recurrences involving the same strain in the context of new SARS-CoV-2 variants with immune escape potential.

Members of the Gregorio Marañón Microbiology-ID COVID-19 Study Group: Javier Adán-Jiménez, Luis Alcalá, Teresa Aldámiz, Roberto Alonso, Beatriz Álvarez, Ana Álvarez-Uría, Juan Berenguer, Elena Bermúdez, Emilio Bouza, Sergio Buenestado-Serrano, Almudena Burillo, Ana Candela, Raquel Carrillo, Pilar Catalán, Emilia Cercenado, Alejandro Cobos, Víctor Manuel de la Cueva, Cristina Díez, Jose Egido-Balzategui, Pilar Escribano, Agustín Estévez, Chiara Fanciulli, Alicia Galar, M. a Dolores García, Darío García de Viedma, Paloma Gijón, Adolfo González, Helmuth Guillén, Jesús Guinea, Laura Vanessa Haces, Marta Herranz, Martha Kestler, Juan Carlos López, Carmen Narcisa Losada, Marina Machado, Mercedes Marín, Pablo Martín, Javier Martín-Escolano, Andrea Molero-Salinas, Pedro Montilla, Patricia Muñoz, María Olmedo, Álvaro Otero-Sobrino, Belén Padilla, María Palomo, Francisco Parras, María Jesús Pérez-Granda, Laura Pérez-Lago, Leire Pérez, Sandra R. Maus, Elena Reigadas, Carla Margarita Rico-Luna, Cristina Rincón, Belén Rodríguez, Sara Rodríguez, Cristina Rodríguez-Grande, Adriana Rojas, María Jesús Ruiz-Serrano, Carlos Sánchez, Mar Sánchez, Julia Serrano, Pedro J Sola-Campoy, Francisco Tejerina, Maricela Valerio, M. Cristina Veintimilla, Lara Vesperinas, Teresa Vicente, Sofía de la Villa.

Appendix 1 Additional data about systematic genomic and clinical analysis of severe acute respiratory syndrome coronavirus 2 reinfections and recurrences involving the same strain

Appendix 2 Additional information about systematic genomic and clinical analysis of severe acute respiratory syndrome coronavirus 2 reinfections and recurrences involving the same strain
