# Supplementary material for: Systematic Genomic and Clinical Analysis of Severe Acute Respiratory Syndrome Coronavirus 2 Reinfections and Recurrences Involving the Same Strain
Source: Emerg Infect Dis. 2022 Jan;28(1):85–94. doi: 10.3201/eid2801.211952 (PMC8714233; doi:10.3201/eid2801.211952)
Supplement: Appendix 2 — Additional information about systematic genomic and clinical analysis of severe acute respiratory syndrome coronavirus 2 reinfections and recurrences involving the same strain [file 21-1952-Techapp-s2.pdf]

Article DOI: https://doi.org/10.3201/eid2801.211952

Systematic Genomic and Clinical Analysis of Severe Acute Respiratory Syndrome Coronavirus 2 Reinfections and Recurrences Involving the Same Strain

Appendix 2

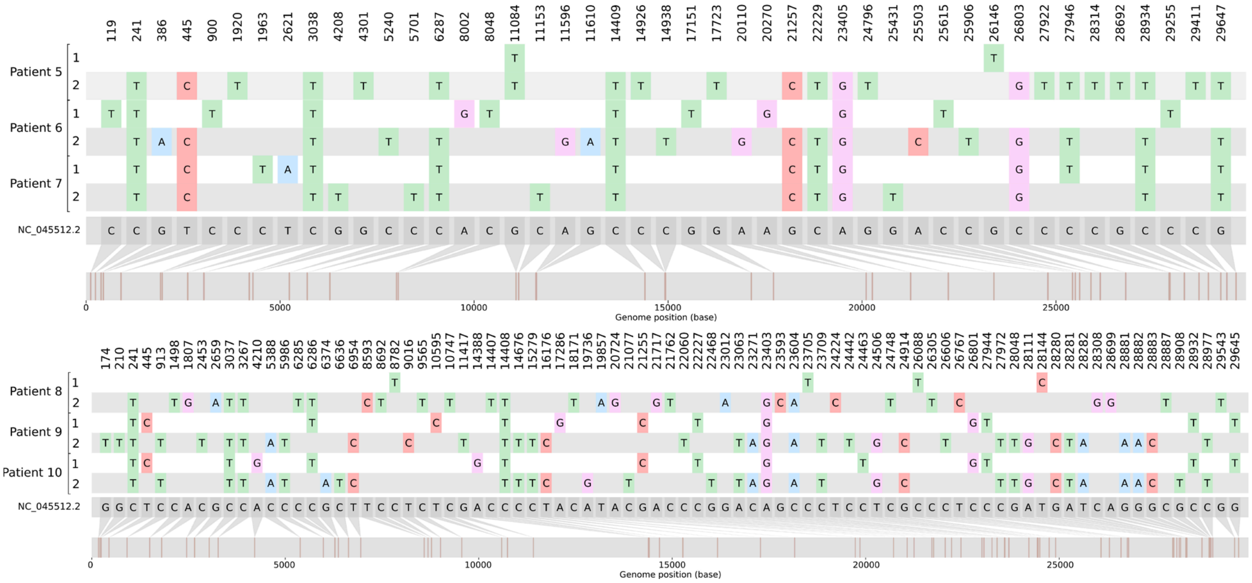

Appendix 2 Figure. Distribution of single-nucleotide polymorphisms in the severe acute respiratory syndrome coronavirus 2 chromosome identified in the 1-to-1 comparison of sequences for the cases (patients 5–10) with both the first (indicated by number 1) and second (indicated by number 2) specimens sequenced and validated by short tandem repeat host analysis to belong to the same person. Indels are not represented in the figure. The sequence NC_045512.2 was used as a reference for Wuhan-1.
